# Prominent peaks and social discourses of the 2023 Escalation in the Ongoing Israel–Hamas armed conflict

**DOI:** 10.1371/journal.pone.0332746

**Published:** 2025-10-09

**Authors:** Reuben Ng, Ting Yu Joanne Chow

**Affiliations:** 1 Lee Kuan Yew School of Public Policy, National University of Singapore, Singapore, Singapore; 2 Lloyd’s Register Institute for the Public Understanding of Risk, National University of Singapore, Singapore, Singapore; The University of Edinburgh, UNITED KINGDOM OF GREAT BRITAIN AND NORTHERN IRELAND

## Abstract

**Background:**

This paper investigates the sharp increase in media posts and engagement surrounding the initial four months (October 2023–January 2024) of the Israel–Hamas armed conflict, following the inciting incident of a surprise militant attack launched on 7 October 2023. The impetus for documenting the trajectory of social media conversations lies in capturing and cataloging the biggest drivers of engagement, public sentiments and groundswell themes, reflecting the public zeitgeist during a period of uncertainty.

**Objectives:**

Few big data studies have delved into initial public discourse surrounding the escalation of the ongoing conflict. First, we identify the biggest generators of buzz, proxied by spikes in mention-counts; secondly, we identify content trends proxied by quantitative sentiment valence, top keywords and emojis, and qualitatively outline the biggest generators of media engagement via top engagement metrics (likes, reposts).

**Methods:**

We analyse a large corpus of publicly-available content from online platforms (Twitter, Reddit, Tiktok) obtained using academic-level API access, containing search terms: *Palestine, Palestinian(s), Israel(i)(s), Gaza, Hamas*. Our first research aim utilizes a prominent peaks model (upper-quartile significance threshold of prominence>1,500,000). Our second research aim utilized qualitative analysis on valence, top keywords and emojis, and top themes.

**Results:**

Eight prominent peaks were identified, finding that news about violence (e.g., airstrikes, citizen harm), groundswell movements (e.g., international activism like worldwide strikes, protests and marches, awareness movements, and outrage in response to current conditions) and politically-charged happenings (e.g., missile strikes) had the biggest hand in boosting discoursal spikes. Valence scores were generally negative, following a general monthly distribution of negative (59%), neutral (31%), and positive (10%), with main keywords focused on terror, violence, and calls for ceasefire. Qualitatively, we find salient groundswell movements (e.g., e-sims for Gaza, content creator strikes for Palestine, circulation of boycott consumer brand lists, co-option of the watermelon emoji as shorthand for support for the cause) and find that the online space is dominated by a fixation on celebrity opinions on the conflict and the circulation of gory footage.

**Conclusions:**

Overall, emergent public chatter worryingly peaks in response to incendiary news about violence, gory footage and celebrity opinions, though discoursal spikes are also slanted toward groundswell movements of goodwill.

## Introduction

Long-standing strife plaguing the Gaza strip has been a major source of humanitarian concern, prompting decades of discursive dissection through various lenses of political science, history, and international law. The region’s conflict stems from deep-rooted tensions over the past century, competing national claims, colonial legacies, and cycles of displacement and occupation, positioned within the broader context of international geopolitics [1] and a protracted struggle over land, sovereignty and identity [2], alongside historical justice [3]. This paper contributes to existing literature from a communications perspective, adding to a robust pool of academic papers that have examined the conflict through the lens of social media. We provide a macro-overview of the most recent wave of the Israel-Hamas armed conflict, engendered by a surprise militant attack launched on 7 October 2023, resulting in the deaths of a reported 1,195 people [4], with further impacts compounded in destroyed infrastructure, severe shortage of essentials and an inability to flee to safety [5].

Few big data studies have covered this most recent onslaught of violence in the region; notably, an investigation into forum posts has yielded a marked increase in controversial comments, intensified discourse, and engagement metrics during this period [6]. Retrospective studies on online discourses covering past key events all point to the significance of social media in mobilizing international audiences and inciting digital action. For instance, various investigations into the 2012-period of the conflict find that shifts in public support on twitter reduce conflict intensity [7]; with image-based visual propaganda frames (resistance, unity, civilian casualties) co-opted to spur emotional engagement [8]. Analyses of the 2014-period of the conflict found that Israel news media primarily framed social media as spaces of hate speech and distribution of rumors; rarely portrayed as platforms to orchestrate collective action [9]; tweets from the Israeli MFA during the 2014 Gaza War co-opted linguistic frames to legitimize Israel’s policies (e.g., images to support strategic narratives of shared identities) [10]. On the flipside, other researchers found that social media itself has become the news [11]: when a celebrity expresses online sympathy with the Palestinian civilians in Gaza [12], or when the Palestinian militant organizations falsify the Israeli army story about taking civilians as human shields [13], or when the supporters of the Palestinians attack Israeli websites and media forums [14], turning the online space into a center for message transmission and the winning of support, sympathy, and public opinion domestically and internationally [15]. In that vein, analyses of the 2022-period of the conflict found that pro-Palestine accounts often heavily tweet about Israeli aggressions, constructing a positive representation of the Palestinian Self and a negative representation of the Israeli Other; a mediated discourse conveyed to international and external audiences [16].

Against this backdrop underscoring the role of social media in mediating and mediatizing conflict, we aim to analyze the initial key narratives that captured public attention during the latest Israel-Hamas armed conflict. We interrogate a corpus dataset of all English-language media content, obtained using academic-level API access across social media platforms (Twitter, Reddit, Tiktok), interrogating content containing search term keywords associated with the conflict (*Palestine, Palestinian(s), Israel(i)(s), Gaza, Hamas*) from October 2023 to January 2024 (n = 1,079,676,984) to ask two main research questions. First, *what events were the biggest generators of buzz, proxied by mention count spikes?* Second, *what content trends can be evinced, using (i) quantitative tracking of sentiment valence, top keywords and emojis, and (ii) qualitative themes found across the biggest generators of media engagement topics, proxied by engagement metrics (likes, reposts)?* Our first aim was achieved by running a prominent peaks model, identifying peaks falling in the upper-quartile significance threshold of prominence (P > 1,500,000), and identifying events associated with these spikes in mention count metrics. Our second aim conducted content analysis in quantitative metrics of valence, top keywords and emojis, and qualitatively identified a subset of highest-engaged posts, delving into the themes that generated the most discussion online, providing a bird’s eye view of the stories that captured the most online attention during this period.

## Methods

### Dataset

**Fig 1:**
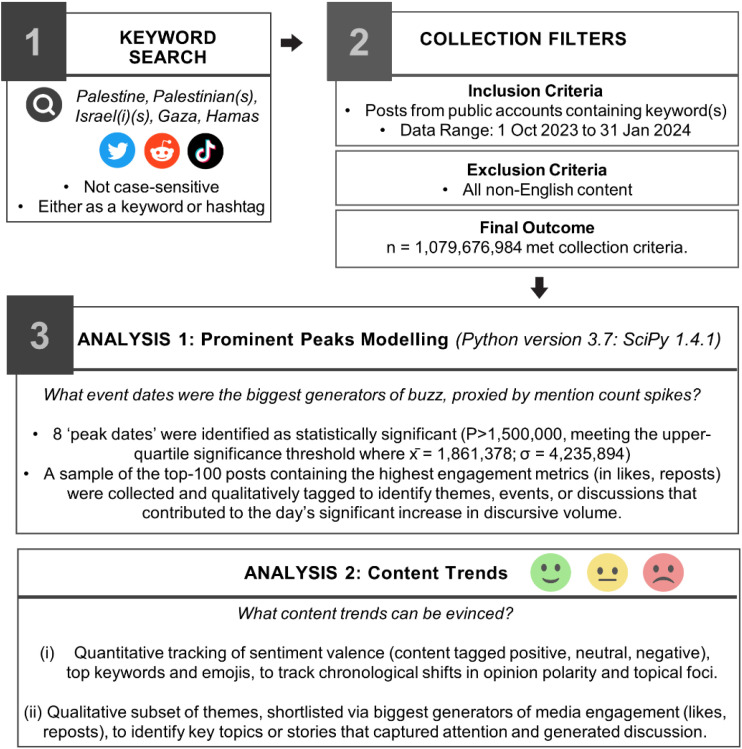
Methodological Summary: Data Collection and Analytic Plan.

The dataset was created by collecting social media data across publicly-accessible textual content across Twitter (using its application programming interface standard search, publicly accessible via API V2 on the academic research access level), Reddit (using the Pushshift Reddit API adhering to fair use non-commercial academic research) and Tiktok (using the Tiktok academic research tools API). All English-language social media content containing one or more the following search term keywords: *Palestine, Palestinian(s), Israel(i)(s), Gaza, Hamas*, used in context as either a word or hashtag, and not case-sensitive, published between October 2023 to January 2024 were shortlisted (n = 1,079,676,984). We provide a summary of methods in Fig 1.

### Research aim 1: prominent peaks modelling

First, we quantitatively identified prominent peak dates which experienced a spike in mention count—defined by the total number of original posts, articles, replies, and retweets containing the search term keywords—signaling a significant increment in interest and engagement in the topic. This analysis was conducted using peak prominence detection modelling (Python 3.7: SciPy 1.4.1). This Python package module operates on marking the higher of two bases as the peak’s lowest contour line; prominence calculated as the vertical difference between the peak’s height, and its lowest contour line. Essentially, the program checks for each given date’s search term keyword mention-count value (A), the lowest possible point *prior* to that date (B) and *after* that date (C): the average of these values (A-B; A-C) is defined as the date’s prominence score, a value assigned relative to the lowest possible point on either side of the chronological calendar.

A total of 8 statistically significant peaks were found during the study period ($\bar{x}$ = 1,861,378; σ = 4,235,894); of which, prominence scores of said peaks were calculated to range from 119,321 (lower quartile), to 449,288 (median), to 1,327,436 (upper quartile). Using a data-driven approach to determine an adequate threshold for significance, we selected peaks that appeared only in the upper quartile of prominence (i.e., P > 1,500,000) for further investigation, essentially, delving into the top 75% of discussion spike dates. This method yielded 8 peaks: 8 individual dates where prominence exceeded the upper quartile’s significance threshold.

**Fig 2:**
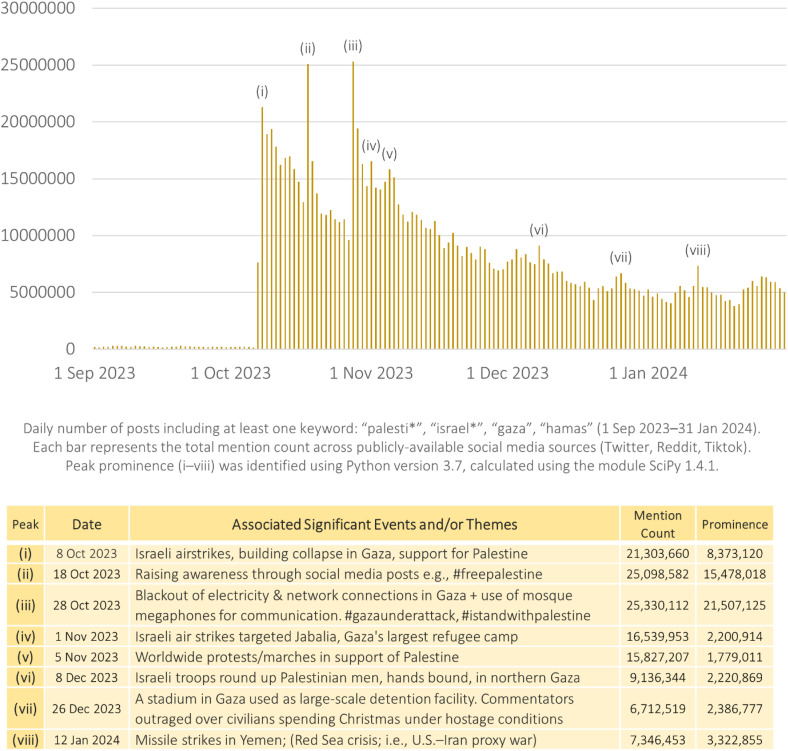
Prominent Peaks of Israel-Hamas Conflict (2023) across 1 billion online posts.

Using these identified 8 upper-quartile peak dates, our second methodological step involved qualitatively sampling the top-engaged posts published on those days (i.e., a qualitative interrogation of their associated events and discussions, using a sample of the top-100 posts containing the highest engagement metrics), to identify the main themes that contributed to the day’s significant increase in discursive volume. A summary of results is presented in Fig 2.

### Research aim 2: content trends

**Fig 3:**
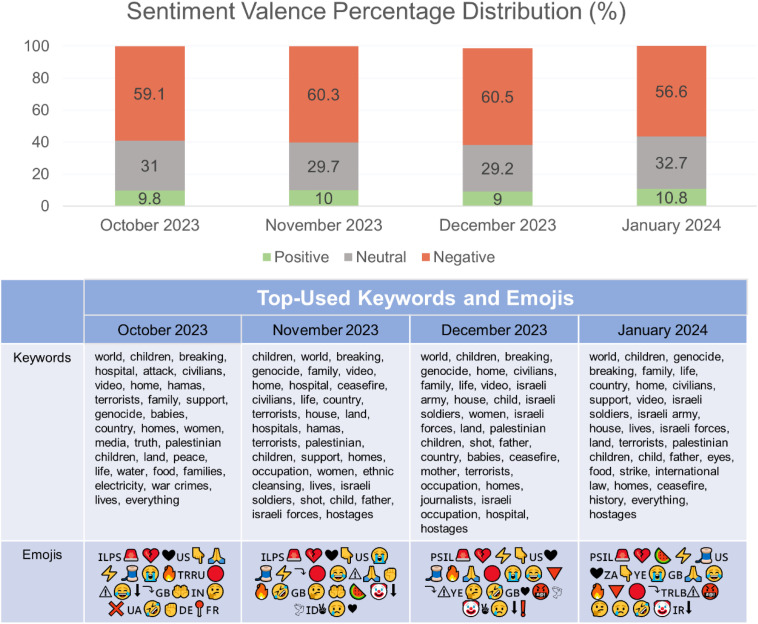
Valence distribution, top keywords and emojis over 4 months of the conflict.

Our second research aim utilized (i) Quantitative tracking of sentiment-tagging to elicit the valence scores associated with each month (October, November, December 2023, January 2024), categorizing the articles as either predominantly negative, neutral, or positive. This was conducted using a transformer-based sentiment model, a natural language processing methodology that was adapted from Google’s open-source pre-trained Bidirectional Encoder Representations from Transformer (BERT) model, which allows for deep bidirectional representations from unlabeled text through joint conditioning left and right context [17]. Each post’s polarity (positive, negative, or neutral sentiment, on the document-level) is generated through this classification method, yielding supervised sentiment-analysis modelled data [18]. Alongside that, we present the highest-frequency keywords and emojis utilized each month. Top keywords were identified by raw count with the manual exclusion of stop-words, defined as auxiliaries containing little-to-no semantic information and serving grammatical functions—for example, determiners (*the, a, an, his, her, this, that),* prepositions (*from, to)* and conjunctions (*and, nor, yet, for, but, or, so*) were omitted from qualifying for this list of highest-frequency keywords. Top emojis were identified by raw count attested in the dataset, and presented in order of highest count. These findings are presented in Fig 3 to provide a bird’s-eye overview of the unfolding conflict. (ii) Qualitatively, we further interrogated a subset of the highest-engaged (likes, reposts) social media posts across Twitter and Tiktok, delving into the main themes that generated the most online discussion and interactions. These qualitative findings are detailed in Table 1.

**Table 1:** Top Qualitative Themes.

Theme	Description
**#1 Celebrity Fixation**	Overwhelmingly, the highest-engaged posts related to this conflict were centered around whether celebrities had posted their opinions on this issue, and the contents of their coverage. Social media users kept track of what their favorite celebrities had said, and detractors of their stand were lambasted for their opposing views. Celebrities who did not post anything were criticized for their silence, as they were deemed responsible to speak on the topic owing to their wide audience and reach. Examples of highest-engaged celebrity posts: • justin bieber posting “praying for israel” using a picture of a destroyed gaza is actually insane https://t.co/GNcEyhNk6V [twitter, Oct 2023] • “I’m not afraid to lose modeling jobs and I will continue to speak up on Palestine” - Bella Hadid #bellahadid [tiktok, Oct 2023] • so noah is too young to be criticized but palestinian children are old enough to die [In response to “I feel bad for Noah Schnapp/the way he’s treated,it’s undeserved. Others with same view on politics don’t get the amount of hate/backlash he gets (most still loved),people pile onto the 19 year old who’s still a teen,it’s disgusting. Genuinely hope he’s okay 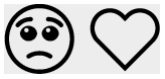 ”] [twitter, Jan 2024]
**#2 Groundswell Support Movements**	Popular streamers and content creators participated in #StrikeForPalestine, placing their streams on hiatus to redirect audiences to sites like https://ceasefiretoday.com/, or conducting their own fundraising events. Common hashtags were: #free_palestine #prayforpalestine #pray_for_gaza #gaza #savepalestine #freepalestinePS #fromtherivertotheseapalastinewillbefree #lovepalestine #fyp #foryoupage #palestine #watermelon speakup #FeedNorthGaza #TreatGazaChildren #GazaCeaseFireNow
**#3 E-sims for Gaza**	In a bid to provide communications within the area, projects like #ConnectingGaza @connectinghumanity_ (Instagram handle) have appealed for donations to fund e-sims access. Several websites dedicated to donating items for relief efforts (food, funds for displaced families, hygiene kits) have also appeared in the form of hyperlinks.
**#4 Circulation of Gory Footage**	Violent videos and images of civilians being kicked, shot, beaten and attacked circulated and often gained the most traction in engagement. • Israeli military tanks in the middle of the Gaza Strip. O Allah, give them strength and safety 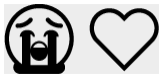 #freepalestine #stopgenocideingaza #gaza [titok, Nov 2023] • 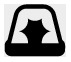 GAZA: HUNDREDS OF PALESTINIANS DETAINED WITHOUT CLOTHING | ON THE GROUND WITH HANDS BOUND Israeli forces are seen detaining Palestinian men, almost naked and hands bound. [twitter, Dec 2023] • Palestinians are now starving to death [tiktok, Dec 2023] • It’s about life or death now I did what I could We are surrounded by the Israeli tanks [twitter, Dec 2023] • Children of Gaza 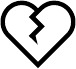 [tiktok, Jan 2024] • The baby girl who remained under the rubble all night in Gaza has made it safely. I see no one sharing. If she were dead, everyone would share! Share positivity... [twitter, Jan 2024] • A 16 year old Palestinian girl had to have her leg amputated by her father on a kitchen table with no anesthesia. [twitter, Jan 2024]
**#5 Boycott List:**	Groundswell chatter led to calls for boycotting consumer brands identified as pro-Israel (e.g., Starbucks, McDonalds), often citing BDS, a two-decade-old movement [Boycott, Divestment, Sanctions] by targeting businesses and institutions accused of aiding violations of Palestinian rights. • Quick update: yesterday night Israel bombed a maternity ward in Khan Younis. We do not need Mcchicken nuggets that bad. #windowstothewall #watermelon #freefalesteen [tiktok, Dec 2023]
**#6 Reminder that the conflict has been ongoing**	Posts re-emphasized that the recent spate of conflict was not the first of its kind, and re-circulated old posts from the region. For example: • I repeat, this did not start October 7th [Repost 4 Jul 2015]: This boy has found his cat alive in the ruins of his home in Gaza. http://t.co/vZk9odRZqq [twitter, Nov 2023]
**#7 Human Interest Story**	• Motaz Azaiza, the photojournalist who documented Israel’s war on Gaza for 107 days through social media, says he feels guilty for being in a safe place after recently leaving the strip. [tiktok, Jan 2024] • Motaz Azaiza: “So, I had to evacuate for a lot of reasons you all know some of it but not all of it. Thank you all Pray for Gaza.” [twitter, Jan 2024]
**#8 Various Commentaries on Violence and Calls for Peace**	• Sorry to see what’s happening in Israel. I hope there can be peace one day. [twitter, Oct 2023] • All Americans should be horrified and outraged by the brazen terrorist attacks on Israel and the slaughter of innocent civilians. We grieve for those who died, pray for the safe return of those who’ve been held hostage, and stand squarely alongside our ally, Israel, as it dismantles Hamas. As we support Israel’s right to defend itself against terror, we must keep striving for a just and lasting peace for Israelis and Palestinians alike. [twitter, Oct 2023] • Israel-Hamas War: 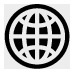 Aftermath of Israeli Defence Forces’ airstrikes on Gaza city in response to Hamas unprecedented attack on Israel. [tiktok, Oct 2023] • No. Sorry. They are not “collateral damage” they are human beings who happen to have been born there and live there and most of those human beings are stuck there. Have some compassion, they are Palestinians not buildings or roads or things, they are human beings and so are the hostages whose lives you may also be destroying. They aren’t “collateral damage” either. [twitter, Nov 2023] • Yesterday I spoke with a Jewish Israeli about why it was important for him to be at the protest. His parents were the only survivors of from the holocaust. He said, “I’m not going to support gen0cide, am I?“#stopgenocideingaza [tiktok, Nov 2023] • i beg you all. while it’s important to talk about palestine, please don’t ignore what’s happening in congo. congo barely has any media coverage, we need to talk about it more. [twitter, Dec 2023] • Joe Biden’s own staff protesting outside the White House, demanding an end to Israel’s genocide in Gaza. [twitter, Dec 2023] • Ronald Lamola outlined the country’s genocide #case against Israel, as a landmark hearing opened at the #internationalcourtofjustice. #news [tiktok, Jan 2024]

## Results

### Prominent peaks

We present findings identifying 8 prominent peaks and emergent events garnering the most mentions within the first four months of the conflict in Fig 2. Peak prominence was observed in dates (i)–(viii), with each spike attributable to a new dominant discoursal theme or event that sparked further discussion.

Detailed in Fig 2, we find that peaks were predominantly driven by three thematic thrusts. First, violence-based happenings spurred mention-count engagement, particularly in news about airstrikes [(i) and (iv)] and news about the rounding up of citizens (vi). Second, metrics were boosted when met with groundswell support in terms of international activism, particularly in awareness movements (ii), citizen outrage on current conditions [(iii) and (vii)], and worldwide strikes, protests and marches (v). Third, politically-charged happenings (viii) also had a hand in boosting discourse and discussions.

### Content trends

#### (i) Quantitative: valence, top keywords and emojis.

Detailed in Fig 3, we find that the main keywords throughout the conflict was focused primarily on the lexical field of war and terrorism (*terrorists, genocide, war crimes, attack, ethnic cleansing, Israeli soldiers/forces, hostages, occupation, shot*), with a semantic focus on pleas for peace (*ceasefire, support, peace, strike, international).* Strong focus was placed on mediatized forms of evidence, particularly owing to various media (videos, pictures) of violence circulating (*video, journalists*)*.* Commentary also places focus on innocent civilians (*children, home(s), family, families, women, lives, father, civilians, house,*) and untold destruction of daily lives (*hospital, land, food, electricity*).

Present in the dataset of top emojis emerged the watermelon emoji (
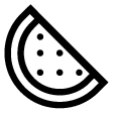
), used as symbolic shorthand for Palestinians/Palestine—owing to the visual containing of the same colors as the flag– to euphemistically avoid censorship on social media sites. This was often used due to the fear of being shadow-banned or deprioritized by the algorithm if the actual term ‘Palestine’ or flag emoji was used, and also used to signal solidarity toward Palestinians. Top emojis also painted a visual reflection of reactions to the ongoing conflict, depicted by both flags (IL,PS) and flags of other nations, often adopted by citizens expressing their international support and discursive engagement in the conflict (US,TR,RU,GB,IN,UA,DE,FR,ID,YE,ZA,LB,IR). Emojis denoting information resources, often using pointing or directional cues were also frequent (
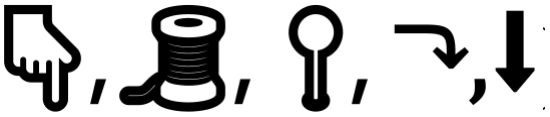
) alongside visual cues on the state of emergency (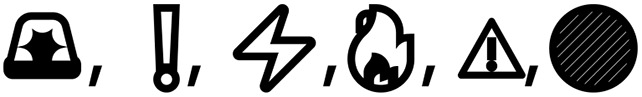). Emojis denoted empathy in emotions like sadness and anger (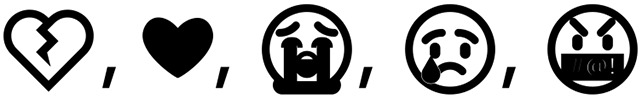), solidarity (
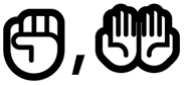
) and calls for peace (
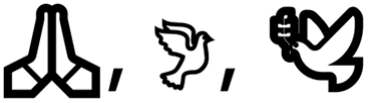
), though a handful depicted derision or making light of the situation (
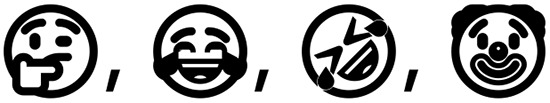
).

#### (ii) Qualitative: highest-engaged themes and topics.

Detailed in Table 1 were, in no particular order of importance, the highest-engaged themes and topics alongside several examples of the discussions around these topics. These posts received the most likes, comments and retweets in the online social media space, reflecting several trends in prevalent groundswell movements.

## Discussion and conclusion

We analyzed large-scale data of emergent public chatter, focusing on engagement and frequency metrics surrounding the most recent spate of conflict. Our research findings have focused on discussions around social media involvement and engagement trends, finding a worrying inclination toward news of violence (e.g., airstrikes, citizen harm) the circulation of gory footage. This trend is significant against a backdrop of growing research in the representation and circulation of violence in social media. Digital technologies have facilitated real-time sharing of unfolding of acts of terror to a global audience, resulting in concerns over the mainstreaming of horrific violence [19] as cyberspace ecologies become more complex [20]. The fact that violence topics garnered the most attention, engagement and discussions is double-edged [21], as increased awareness and exerts active political pressure [22], yet its psychological impact on unwitting viewers may lead to distress and anxiety [23]; and at times confusing, should its context of origin be unsubstantiated This presents impetus for policymaker attention, given that the proliferation such imagery represents an ambivalent phenomenon in digital culture: while emotionally-charged moralized content diffusing through social networks are potentially polarizing [24] and psychologically impactful [25], its very circulation represents a kind of informational mobility that often bypasses gatekeepers like traditional media or government institutions, to serve as unfiltered representations of lived realities. They can bear witness to atrocity, force acknowledgment of harm, and galvanize public attention and political action in ways that sanitized representations cannot. In this sense, their virality may function as a form of grassroots documentation or testimony within its discursive contexts.

Our findings also reveal that engagement and mention metrics were skewed toward celebrity opinions. This validates a growing body of research into celebrity influence on social advocacy, with findings suggesting that expected scopes of traditional entertainment have expanded to encompass broader global politics and domains of international diplomacy [26] and activism [27], leveraging their large audience bases to further social causes, with professional repercussions for stands taken [28]. The fixation on celebrity stances is emblematic of digitized conflict advocacy in the current century [29], which has political impacts in influencing perceptions, especially amongst the youth [30], through endorsements of social issues [31] and causes on social media platforms [32]. The prioritization of celebrity opinions attested in this study therefore reinforces and adds to the body of literature investigating political opinions in the mediatized context.

The negativity inherent in the dataset topic is countered by discoursal spikes and engagement metrics represented by groundswell movements (e.g., international activism like worldwide strikes, protests and marches, awareness movements, and outrage in response to current conditions) and actionable items (e.g., e-sims for Gaza, content creator strikes for Palestine, continued circulation of boycott consumer brand lists, co-option of the watermelon emoji as shorthand for support for the Palestinian cause). Significantly, the dominance of Palestinian groundswell causes and narratives focused on human rights violence and violations shapes perceptions on a global scale, attesting to long-standing unified strikes [33] and mobilization, even from international non-diasporic communities [34]. This amplification of specific narratives to evoke emotional empathy [35] and spur collective action is worth examining from a tactical [36] and message framing perspective [37].

The scope limitations of this study are acknowledged. The macro-level line of inquiry—meant to springboard a broader sociological viewpoint for the biggest topics that led to increased interaction and engagement permeating online spaces—is a limitation which may be complemented with a micro-level approach studying individual platforms, or the inclusion of other social media spaces like Facebook and Instagram, or broadcast media across news outlets and official distribution channels. Future research may involve tracking the chronological progression of this conflict, adopting the replicability of the methodology [38]. By that token, the focus on macro-level trends across 1 billion posts does not account for micro-level narratives, individual actions, and small-scale activism and storylines that cover greater depth and detail. We further acknowledge that social media affordances and ecologies vary across platforms: there exist differences in degrees of anonymity and network association [39]. Follow-up studies may consider micro-interactions on a smaller scale to decipher the nuances of conversational content on individual platforms. Furthermore, search term keyword data was more commonly attested on text-heavier platforms of Twitter and Reddit compared to Tiktok, where video creators speaking on these topics may not have used the search term keywords in caption data. Complementary studies may consider qualitatively coding short-form video content to elicit these embedded discussions.

Lastly, a limitation of this study is that the search term keywords (*Palestine, Palestinian(s), Israel(i)(s), Gaza, Hamas*) do not represent the full scope of the conflict, as many users have taken to deliberately misspelling these search terms, employing coded language, or using symbolic representations (e.g., the watermelon, as discussed) to evade platform censorship or moderation flagging [40]. Further, while the study’s data collection phase occurred in context of full firehose data obtained via academic-level access, posts available for analysis were strictly derived from public accounts, thus representing a subset of viewpoints across all users, to the exclusion of private accounts who may also have added to the discussion in personal capacities.

Overall, our study provides a broad-based macro-overview of the biggest topical buzz generators in the social media landscape, foregrounding key insights on emergent public chatter, adding to the repository of literature documenting the conflict and its global implications.
